# Copper Modulates the Catalytic Activity of Protein Kinase CK2

**DOI:** 10.3389/fmolb.2022.878652

**Published:** 2022-06-09

**Authors:** John E. Chojnowski, Rongrong Li, Tiffany Tsang, Fatimah H. Alfaran, Alexej Dick, Simon Cocklin, Donita C. Brady, Todd I. Strochlic

**Affiliations:** ^1^ Department of Biochemistry and Molecular Biology, Drexel University College of Medicine, Philadelphia, PA, United States; ^2^ Department of Cancer Biology, Perelman School of Medicine, University of Pennsylvania, Philadelphia, PA, United States

**Keywords:** casein kinase 2 (CK2), copper, cell signaling, protein kinase (CK2), phosphorylation, kinase regulation

## Abstract

Casein kinase 2 (CK2) is an evolutionarily conserved serine/threonine kinase implicated in a wide range of cellular functions and known to be dysregulated in various diseases such as cancer. Compared to most other kinases, CK2 exhibits several unusual properties, including dual co-substrate specificity and a high degree of promiscuity with hundreds of substrates described to date. Most paradoxical, however, is its apparent constitutive activity: no definitive mode of catalytic regulation has thus far been identified. Here we demonstrate that copper enhances the enzymatic activity of CK2 both *in vitro* and *in vivo*. We show that copper binds directly to CK2, and we identify specific residues in the catalytic subunit of the enzyme that are critical for copper-binding. We further demonstrate that increased levels of intracellular copper result in enhanced CK2 kinase activity, while decreased copper import results in reduced CK2 activity. Taken together, these findings establish CK2 as a copper-regulated kinase and indicate that copper is a key modulator of CK2-dependent signaling pathways.

## Introduction

The human kinome consists of 535 kinases capable of phosphorylating approximately 70% of the proteins expressed in human cells (Olsen et al., 2010; Sharma et al., 2014; Ardito et al., 2017; Sugiyama et al., 2019). Of these kinases, CK2 (formerly known as casein kinase 2) is predicted to be responsible for generating over 20% of the phosphoproteome (Burnett & Kennedy, 1954; Salvi et al., 2009). Due to its significant contribution to the phosphoproteome and the fact that it phosphorylates and regulates the activity of other kinases, CK2 is considered a master kinase. As such, CK2 regulates a host of critical physiologic functions, including cell division, cell survival, gene expression, and protein folding, and has been implicated in the progression of multiple diseases (Litchfield, 2003; Meggio & Pinna, 2003; Borgo et al., 2021; Salvi et al., 2021; Strum et al., 2021).

CK2 is an evolutionarily conserved serine/threonine kinase and is a heterotetramer composed of two catalytic α subunits and two regulatory β subunits. The α subunit can phosphorylate substrates as part of the tetrameric complex or as a monomer (Grankowski et al., 1991). An additional isoform of the catalytic subunit, CK2α’ is expressed in humans, is catalytically active, and shares approximately 90% identity with CK2α in its catalytic domain (Litchfield, 2003). CK2 exhibits dual co-substrate specificity and can use either ATP or GTP to phosphorylate substrates (Niefind et al., 1999). Intriguingly, most studies to date suggest that CK2 is constitutively active and has no defined mode of catalytic regulation, a finding that is bewildering given its central role in the regulation of many core cellular processes (Pinna, 2013).

Despite CK2 being considered constitutively active, numerous studies provide solid evidence of varied enzymatic activity. Most of these reports suggest that these differences are likely due to changes in CK2 expression levels (Strum et al., 2021). However, several other studies document significant alterations in CK2 catalytic activity even when changes in expression and substrate abundance are considered, thus underscoring a likely unidentified mechanism for regulating CK2 kinase activity (Dubois et al., 2016; Purzner et al., 2018). Specifically, evidence from *in vitro* experiments suggests that polybasic molecules, such as polyamines, can increase the catalytic activity of CK2, but inconsistent catalytic enhancement was observed in cell-based assays (Leroy et al., 1997; Lawson et al., 2006; Ao et al., 2019). The binding of inositol phosphates to CK2 can enhance catalytic activity for certain substrates (Solyakov et al., 2004), and CK2 phosphorylation by Src, Lyn, and protein kinase C has also been shown to stimulate CK2 kinase activity (Donella-Deana et al., 2003; Lee et al., 2016). Furthermore, the binding of CK2 to other proteins such as fibroblast growth factor, p21, and Lamin A alters CK2 activity and promotes or inhibits the phosphorylation of specific substrates (Skjerpen et al., 2002; Jia et al., 2016; Ao et al., 2019). However, in general, most of these examples are cell-type- or context-specific, and there is currently no known universal mechanism for the catalytic regulation of CK2 kinase activity (Roffey & Litchfield, 2021).

Recent studies have uncovered a novel role for copper in modulating the activity of specific protein kinases. Copper was first identified as being essential for the activity of mitogen-activated protein kinase 1 (MEK1) and has since been demonstrated to regulate the activity of several other kinases, including Unc-51 like autophagy activating kinase (ULK1) and 3-phosphoinositide dependent protein kinase 1 (PDK1) (Brady et al., 2014; Tsang et al., 2020; Guo et al., 2021). Here, we identify CK2 as a copper-regulated kinase. We demonstrate that copper binds directly to the enzyme, enhancing its catalytic activity both *in vitro* and *in vivo*. These findings expand the repertoire of kinases regulated by copper and suggest that copper-mediated regulation is an important mechanism for modulating CK2 activity.

## Materials and Methods

### Plasmids

CK2 plasmids were a gift from David Litchfield and obtained through Addgene: GST-CK2α (pDB1-CK2α, #27083) and CK2α-HA (pZW6-CK2α, #27086) (Turowec et al., 2010). The CK2α-Met153Ala/His154Ala mutant was generated by site-directed mutagenesis using the QuikChange kit (Agilent). Mutagenic primer sequences are available upon request. All constructs were fully sequenced.

### Metal Ion-Binding Site Prediction

CK2α structures were acquired from the Protein Data Bank (https://www.rcsb.org/). These PDB files were uploaded to the MIB (Metal Ion-Binding site prediction and docking server) (http://bioinfo.cmu.edu.tw/MIB/), and representative images were generated by the server (accessed March 2022).

Predicted copper-binding residues were included if the residues were predicted to bind copper across three independent PDB structures (3war, 3at2, 5zn0) uploaded to MIB, with all other residues excluded that did not fit the criteria. Binding potentials were normalized to the highest scoring predicted copper-binding residue.

### Protein Expression and Purification

Purification of CK2α was performed as described (Turowec et al., 2010). In brief, competent BL21 *E. coli* were transformed with plasmids encoding CK2α-GST WT or CBM. Transformed cells were grown in Terrific Broth (TB) at 37°C until reaching an optical density of 0.6–1.0 (at 600 nM) and then protein expression was induced with 500 µM Isopropyl β-d-1-thiogalactopyranoside (IPTG) overnight at 15°C. Cells were centrifuged at 4,000 rpm for 1 h at 4°C. Bacterial pellet was resuspended in 30 ml of lysis buffer (PBS pH 7.4, 1M NaCl) and then frozen for 24-h at −80°C. Cells were thawed on ice and 30 ml of additional lysis buffer with reagents making the final concentration of 10% TritonX-100, 1 mg/ml lysozyme, and protease inhibitors (Roche). The lysate was sonicated (QSonica) at 70% amplitude cycles for 10 s on and 20 s off pulses for 2 min total on ice. Lysates were ultracentrifuged at 45,000 rpm for 45 min at 4°C. The supernatant was filtered through 20 µm filter and run over a gravity column with 5 ml glutathione agarose (GoldBio). The column was washed with 150 ml of PBS (pH 7.4) containing 1M NaCl, 10 mM EDTA, and 1% Triton X-100. Protein was eluted in 50 mM Tris-Cl (pH 8.0), 1 mM DTT, and glutathione ranging from 10 to 200 mM. For cleavage with Factor-Xa (New England Biolabs), protein underwent buffer exchange using a spin column with a 30 kDa molecular weight cut-off (Corning) into 20 mM Tris-Cl, 100 mM NaCl, 2 mM CaCl_2_. Protein was incubated for 24 h at 4°C and subsequently applied to a gravity column with 3 ml GSH-resin. The flow-through was applied to a fast protein liquid chromatography system with a size-exclusion column HiLoad 16/60 Superdex 200 (Sigma Aldrich). Proteins eluted at the correct size were collected and underwent buffer exchange and concentration by spin column with 30 kDa molecular weight cut off (Corning) into storage buffer (15 mM MOPS pH 7.0, 0.75 mM DTT, 300 mM ammonium sulfate, with 5% glycerol). Following concentration, protein was flash-frozen and stored at -80°C.

### Cell-Based Methods

HeLa, HEK293, A375, and U87 MG cells were obtained from ATCC and maintained in Dulbecco’s Modified Eagle Media (DMEM) supplemented with 10% v/v fetal bovine serum (FBS) and 1% penicillin-streptomycin (P/S) at 37°C in a humidified incubator with 5% CO_2_. *Ctr1* ^*−/−*^ MEFs were generously shared by the Brady Lab (University of Pennsylvania) and maintained in Dulbecco’s Modified Eagle Media (DMEM) supplemented with 10% v/v fetal bovine serum (FBS) and 1% penicillin-streptomycin (P/S) at 37°C in a humidified incubator with 5% CO_2_.

For transient transfection, HEK293 cells were plated into 10 cm plates. The cells were transfected following the Lipofectamine 2000 protocol (ThermoFisher) with pZW6-CK2α-HA-WT or CBM plasmids. Cells were lysed in 100 µL or 500 µL RIPA buffer (50 mM Tris-HCl, pH 7.4, 150 mM NaCl, 0.5% sodium deoxycholate, 1% NP-40) containing 20 mM EDTA and 2X EDTA-free Halt protease and phosphatase inhibitors (Thermo Fisher Scientific). Cells were scraped off the plate and transferred into microcentrifuge tubes. Lysates were subjected to homogenization by passing them twice through an insulin syringe. Samples were subsequently incubated on ice for 30 min, followed by centrifugation at 21,000xg at 4°C for 15 min. Following centrifugation, the supernatant was removed, and the protein concentration was determined by BCA protein assay (Pierce) using BSA as a standard.

For various treatments, HeLa, A375, or U87-MG cells were sub-cultured into a 6-well plate seeded at a density of 150,000 cells per well and allowed to grow for 48 h. After 48 h, cells were washed twice in PBS, and the media was replaced with serum-free media. After 24 h in serum-free media, 1 µM CX-4945 (in DMSO) was added. After 24 h of growth in serum-free media with (or without) CX-4945, 10% serum, 100 µM copper, or 100 µM Cu-ATSM (in DMSO) was added, as indicated. After 1 h of treatment, the cells were lysed as described, followed by SDS-PAGE and immunoblot analysis.

### Immunoblot Analysis

Proteins were resolved by SDS-PAGE and detected using the following primary antibodies: mouse anti-GAPDH (1:4,000 in BSA, #32233, Santa Cruz), mouse anti-CK2α (1:2000 in BSA, #373894, Santa Cruz), mouse anti- CK2α’ (1:100 in BSA, #514403, Santa Cruz), mouse anti-CK2β (1:2000 in BSA, #46666, Santa Cruz), mouse anti-CCS (1:500 in BSA, #55561, Santa Cruz), mouse anti-PTEN (1:500 in BSA, #7974, Santa Cruz), mouse anti-CDC37 (1:1000 in BSA, #4793, Cell Signaling), mouse anti-GST (1:2000 in BSA, #138, Santa Cruz), rabbit anti-ERK (1:1000 in BSA, #4695, Cell Signaling), rabbit anti-phospho-Thr202/Tyr204-ERK (1:1000 in BSA, #4370, Cell Signaling), rabbit anti-MEK1/2 (1:1000 in BSA, #9122, Cell Signaling), rabbit anti-phospho-Ser13-CDC37 (1:1000 in BSA, #108360, Cell Signaling), rabbit anti-phospho-PTEN (Ser380/Thr382/383) (1:500 in BSA, #9549, Cell Signaling), rabbit anti-phospho-CK2 Substrate [(pS/pT)DXE] MultiMab (1:1000 in BSA, #8738S, Cell Signaling), rabbit anti-AKT (1:1000 in BSA, #4691, Cell Signaling), rabbit anti-phospho-Ser129-AKT (1:500 in milk, #13461, Cell Signaling), rabbit anti-thiophosphate ester (1:1000 in BSA, #92570, Abcam), and rabbit-anti-HA (1:500 in BSA, #3724, Cell Signaling). Primary antibody incubation was followed by detection with secondary antibodies: IRDye® 800CW Goat anti-Mouse IgG (1:10,000 BSA, 926-32210, Licor), IRDye® 680RD Goat anti-Rabbit IgG (1:10,000, 926-68071, Licor), or anti-rabbit goat HRP-linked (1:1000, 7074, Cell Signaling) with ImmunoCruz Western Blotting Luminol (Santa Cruz) detection reagent. Immunodetection was performed using infrared imaging on the Odssey CLx (Licor), or horseradish peroxidase, using GeneSys (Syngene). The fold-change in the ratio of phosphorylated protein to total protein was quantified using ImageJ.

### *In vitro* Kinase Assays

Reactions contained either 500 U of heterotetrameric CK2 (New England Biolabs) or 250 nM purified CK2α with copper, TTM, or Cu-ATSM, as indicated. Reactions were incubated with GST-Jabba (250 µM) or GST-AKT (60uM [Abcam ab62279]) and 2 mM DTT in reaction buffer (50 mM HEPES pH 7.5, 0.65 mM MgCl_2_, 0.65 mM MnCl_2_, 12.5 mM NaCl) for 30 min on ice prior to the addition of 500 µM ATPγS, GTPγS, or ATP for 1 min @30°C. Reactions were quenched with 25 mM EDTA. Following the reaction being quenched, 2.5 mM *p*-nitrobenzyl mesylate (Abcam) was added for 1 h at room temperature. Sample buffer was then added to quench the alkylation reaction. SDS–PAGE analysis and immunoblotting were performed as described.

For the immunoprecipitation (IP)-kinase assay, HEK293 cells were transfected as described with pZW6-CK2α-HA WT and CBM plasmids. Cells were lysed as described without EDTA in lysis buffer. Lysates were pre-cleared with 25 µL packed A/G beads (Santa Cruz) for 1 h with rotation at 4°C. Following incubation, lysates were centrifuged at 21,000xg for 5 min at 4°C. Subsequently, 2.5 µL of anti-HA antibody (Cell Signaling) was added to the supernatant and incubated overnight. The next morning, 50 µL of packed A/G beads (Santa Cruz) were added to the tubes for 2 h with rotation at 4°C. Following incubation, samples were centrifuged at 21,000xg for 5 min and washed with 500 µL RIPA buffer three times. Following the final wash, samples were resuspended in reaction buffer (50 mM HEPES pH 7.5, 0.65 mM MgCl_2_, 0.65 mM MnCl_2_, 12.5 mM NaCl) with GST-tagged Jabba (250 µM) and 2 mM DTT. Reactions were incubated with 500 µM ATPγS for 10–30 min @30°C. Reactions were then quenched with 25 mM EDTA. Following quenching, 2.5 mM *p*-nitrobenzyl mesylate was added for 1 h at room temperature. After 1 h, sample buffer was added to quench the alkylation reaction. SDS–PAGE analysis and immunoblot were performed as described.

### Resin-Binding Assays

1000 U of purified recombinant CK2 (NEB) in RIPA buffer containing 50 µL Profinity IMAC resin (Bio-Rad) charged with no metal, Cu^2+^, Zn^2+^, or Fe^3+^, for 1-h at 4°C. Following incubation, samples were centrifuged at 1000xg at 4°C, and the supernatant was discarded. Samples were washed with 500 µL of RIPA buffer and centrifuged at 1000xg three times. Following washes, sample buffer was added. SDS–PAGE analysis and immunoblotting were performed as described.

For resin-binding assays using cell lysate, HEK293 cells were transfected with pZW6-CK2α-HA-WT or CBM plasmids. Cells were lysed as described. 100µg of cell lysate was incubated in binding buffer (RIPA buffer with 20 mM Imidazole) containing 50 µL Profinity IMAC resin (Bio-Rad) charged with no metal, Cu^2+^ for 1-h at 4°C. Following incubation, samples were centrifuged at 1000xg at 4°C, and the supernatant was discarded. Samples were washed with 500 µL of binding buffer and centrifuged at 21000xg three times. Following the three washes, sample buffer was added. SDS–PAGE analysis and immunoblotting were performed as described.

### Enzyme-Linked Immunosorbent Assay (ELISA)

500 U of recombinant CK2 holoenzyme (New England Biolabs) was preincubated with 25 µM copper sulfate and 500 µM DTT in reaction buffer (50 mM HEPES pH 7.5, 625 µM MgCl2, 625µM MnCl2, 12.5 mM) for 45 min on ice. Reactions were then incubated in reaction buffer with 500 µM ATP or GTP on the ELISA plate (CycLex CK2 kit) for 30 min at 30°C. Reactions were quenched with 100 mM EDTA. Following quenching, all steps from the ELISA protocol kit were followed, and samples were read at 450 nM on a plate reader.

### Inductively coupled plasma-mass spectrometry (ICP-MS)

Purified CK2α (22.5 µM) was incubated in the absence or presence of varying concentrations of copper sulfate (22.5µM, 112.5 µM) along with varying concentrations of DTT (112.5µM, 675 µM) at five times the concentration of copper. The buffer used in the reactions was 50 mM HEPES pH 8.0, 150 mM NaCl with 5% glycerol. The reaction occurred on ice for 30 min. Following incubation, samples were applied to a pre-equilibrated PD SpinTrap G-25 column to remove excess copper. Samples were subsequently analyzed by ICP-MS at the Pennsylvania Animal Diagnostic Laboratory System at the University of Pennsylvania School of Veterinary Medicine.

## Results

### Identification of CK2 as a Putative Copper-Regulated Kinase

**FIGURE 1:**
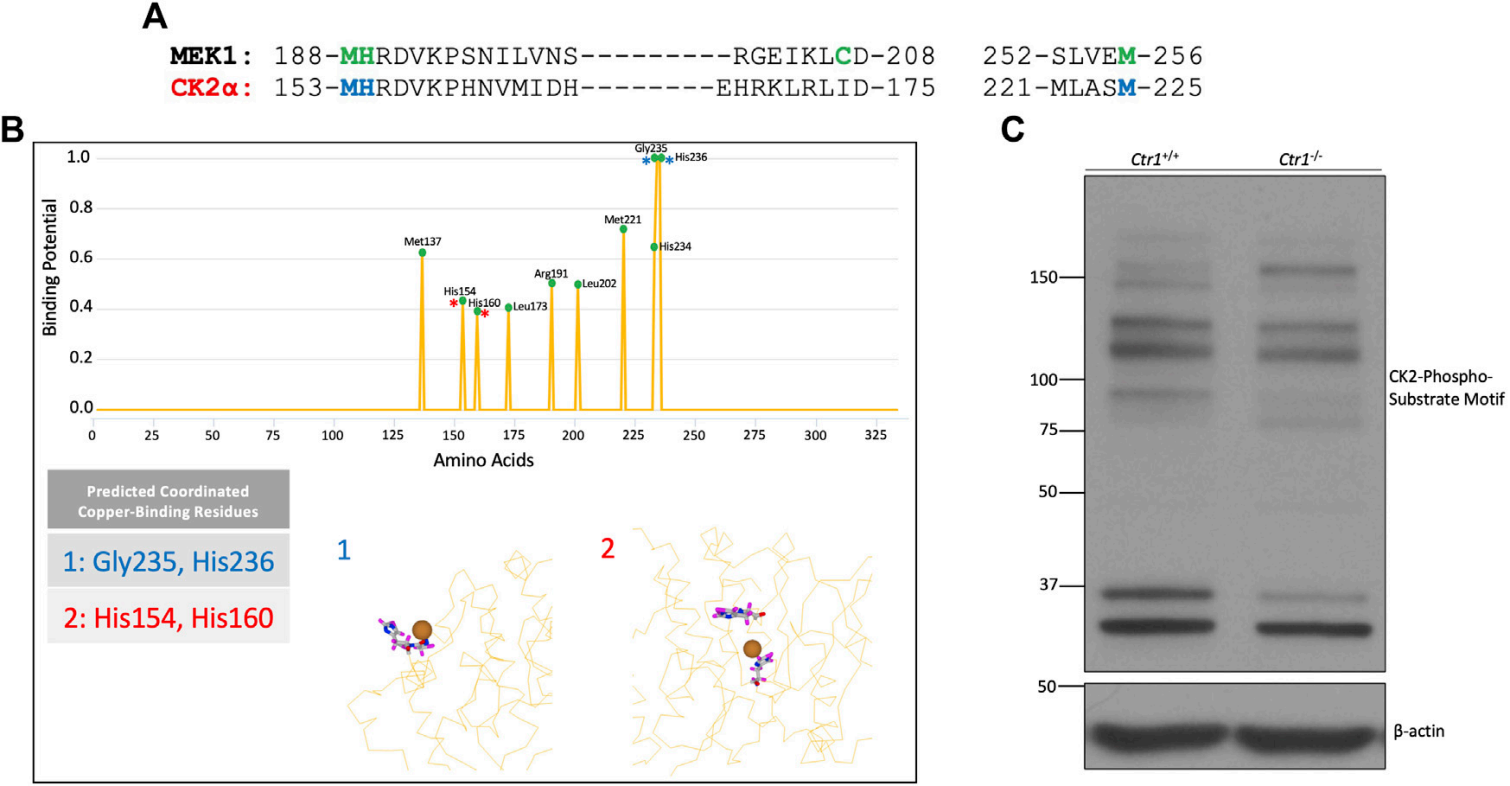
Identification of CK2 as a putative copper-dependent kinase. **(A)** Alignment of the amino acid sequences of human MEK1 and CK2α. The green letters represent amino acids involved in copper-binding by MEK1. The blue letters represent residues in CK2α that share homology with MEK1 at copper-binding residues. **(B)** Prediction output by the MIB site prediction server (Lin et al., 2016) using three PDB files: 3war, 3at2, and 5zn0 (Kinoshita et al., 2011; Kinoshita et al., 2013; Shibazaki et al., 2018). Residues were only included if they were predicted to bind copper across all three structures. The top panel represents the prediction scores across CK2α, with predicted potential binding sites denoted by a green circle. The bottom panel represents two predicted binding sites. Site 1 represents a surface-exposed binding site composed of residues Gly235 and His236, indicated by blue asterisks in the top panel. Site 2 represents a predicted binding site buried in the catalytic domain including residues His154 and His160, indicated by red asterisks in the top panel. **(C)** Immunoblot analysis of lysates from *Ctr1* ^*+/+*^ and *Ctr1* ^−/−^ MEFs probed with the indicated antibodies.

To investigate if CK2 is a putative copper-dependent kinase, we analyzed the homology between CK2α and the established copper-binding kinase MEK1 (Figure 1A). Alignment of the sequences of CK2α and MEK1 confirmed that several of the critical copper-binding residues in MEK1 are indeed conserved in CK2α. Additionally, several of these residues are conserved in two other copper-binding kinases, ULK1 and PDK1 (Supplemental Figure S1). To further explore the potential of CK2 regulation by copper, we took advantage of an in-silico approach: the Metal Ion-Binding Site Prediction and Docking Server (Lin et al., 2016). Through analysis of various CK2α crystal structures in the Protein Data Bank (PDB) (Kinoshita et al., 2011; Kinoshita et al., 2013; Shibazaki et al., 2018), multiple residues predicted to be implicated in copper binding were identified (Figure 1B). The majority of these residues are located within the catalytic domain of CK2α and are identical to the residues identified by homology analysis with MEK1.

Additionally, the server generated predictions as to which residues might be capable of coordinating copper together (Figure 1B). These sites clustered in two major predicted copper-binding sites within CK2α: a binding site on the protein’s surface (site 1) and a binding site within the catalytic pocket (site 2). The latter aligned with the predicted binding based on homology analysis with MEK1 (Figure 1A). Collectively, the results from this analysis suggest that CK2α has the capacity to interact with copper via residues in its catalytic domain.

To validate these findings, we investigated whether endogenous CK2 kinase activity was dependent on the expression of Ctr1, the primary copper transporter in mammalian cells (Lee et al., 2002). Knockout of *Ctr1* has been shown to modulate copper-dependent signaling by both MEK and ULK (Turski et al., 2012; Brady et al., 2014; Tsang et al., 2020). Specifically, we used mouse embryonic fibroblasts (MEFs) in which the gene encoding Ctr1 was deleted to examine if reduction in copper import influences global CK2 signaling. We assessed CK2 signaling by Western blot analysis using a pan-phospho-CK2 substrate motif antibody that demonstrated differential banding when comparing lysates from *Ctr1* ^*+/+*^ versus *Ctr1* ^−/−^cells, suggesting an underlying difference in CK2 substrate phosphorylation dependent on intracellular copper status (Figure 1C). Together, these data suggest that CK2 is a putative copper-regulated kinase.

### Copper Enhances CK2 Kinase Activity *in vitro*

**FIGURE 2:**
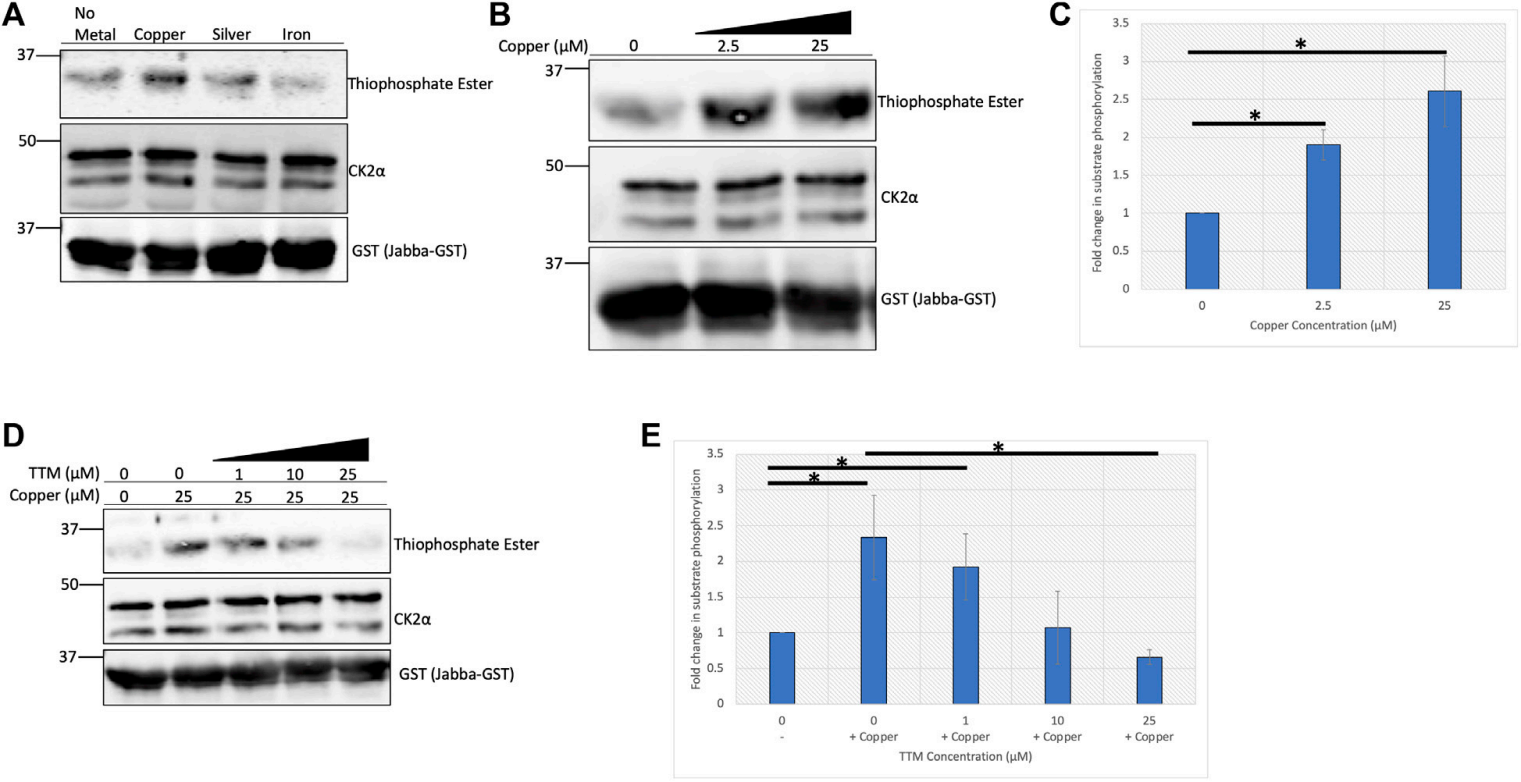
Copper enhances CK2 activity *in vitro*. **(A)** *In vitro* kinase assay assessing CK2 activity in the presence of various metals. Recombinant heterotetrameric CK2 was incubated with GST-tagged Jabba and ATPγS in the presence or absence of the indicated metals (25 µM). Following incubation with PNBM, reactions were resolved by SDS-PAGE and analyzed by immunoblotting with the indicated antibodies. **(B)** *In vitro* kinase assay assessing CK2 activity in the presence of increasing concentrations of copper. Recombinant heterotetrameric CK2 was incubated with GST-tagged Jabba and ATPγS in the presence or absence of copper. Following incubation with PNBM, reactions were resolved by SDS-PAGE and analyzed by immunoblotting with the indicated antibodies. **(C)** Quantification of the thiophosphate ester signal detected by immunoblot analysis in panel B (n = 4). Data are represented as mean fold change ±SEM. *indicates *p* < 0.05 as calculated by a paired student *t*-test. **(D)** *In vitro* kinase assay assessing CK2 activity in the presence of copper and increasing concentrations of TTM. Recombinant heterotetrameric CK2 was incubated with GST-tagged Jabba and ATPγS in the presence or absence of copper and various TTM concentrations. Following incubation with PNBM, reactions were resolved by SDS-PAGE and analyzed by immunoblotting with the indicated antibodies. **(E)** Quantification of the thiophosphate signal detected by immunoblot in panel D (n = 3). Data are represented as mean fold change ±SEM. *indicates *p* < 0.05 as calculated by a paired student *t*-test.

In order to directly assess the influence of copper on CK2 enzymatic activity, we performed an *in-vitro* kinase assay. A previously validated substrate of CK2, Jabba, was incubated with recombinant CK2 holoenzyme and ATPγS in the presence or absence of copper or other metals, allowing for thiophosphorylation of the substrate (McMillan et al., 2018; Chojnowski et al., 2019). Subsequent addition of the alkylating agent *p*-nitrobenzyl mesylate (PNBM) generates a thiophosphate ester moiety that can be detected with an anti-thiophosphate ester antibody by Western blotting. Copper, and to a lesser extent silver (which is isoelectric to cuprous copper), increased CK2 kinase activity as evidenced by increased phosphorylation of Jabba, whereas iron had no apparent effect (Figure 2A). Due to the observed increase in CK2 activity when incubated with copper, a titration experiment was performed that demonstrated a dose-dependent copper-mediated 2.5-fold enhancement in CK2 activity (Figure 2B,C). Copper-mediated enhancement of CK2 activity was confirmed in an independent ELISA assay using p53 as the substrate (Supplemental Figure S2). Interestingly, the enhancement seen with ATPγS or ATP was not observed when GTPγS or GTP was used in either the *in vitro* reaction or the ELISA (Supplemental Figures S2, S3), suggesting that the effect of the metal is nucleotide-specific.

To further investigate the enhancement of CK2 activity by copper, increasing concentrations of the copper-specific chelator tetrathiomolybdate (TTM) were titrated into the kinase reaction (Ogra et al., 1996). As the concentration of the chelator approached equimolar concentrations of copper in the reaction, the enhancement in CK2 activity was abolished and decreased to baseline (Figure 2D,E), and we confirmed that incubation with TTM alone had no effect on CK2 activity (Supplemental Figure S4). Together, these results indicate that cuprous copper enhances CK2 kinase activity *in vitro* and that copper chelation abrogates this effect.

### CK2 Binds Directly to Copper

**FIGURE 3:**
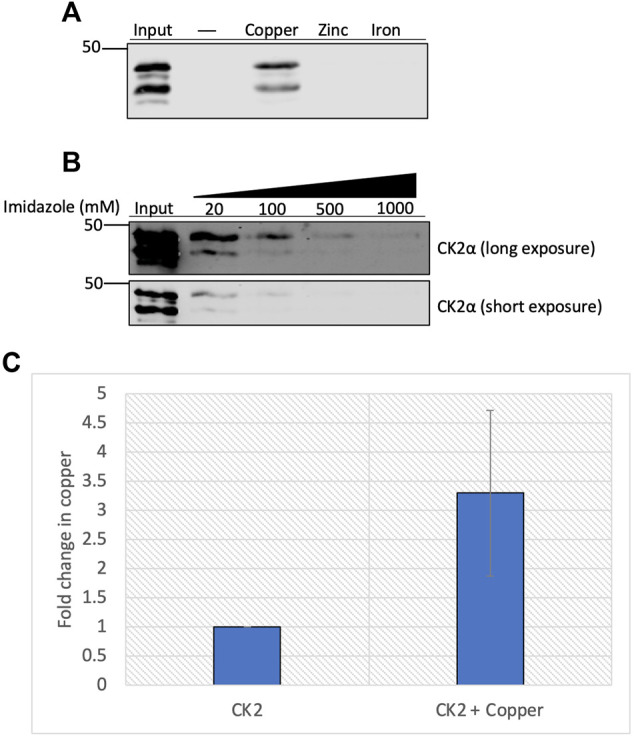
Copper binds directly to CK2α. **(A)** Resin binding assay assessing the interaction of CK2 with various metals. Recombinant heterotetrameric CK2 was incubated with resin charged with or without copper, zinc, or iron. Input represents 20% of total protein incubated with each resin, respectively. Eluted protein was resolved by SDS-PAGE and assessed by immunoblotting with the indicated antibodies. **(B)** Competition assay assessing the capacity of imidazole to compete for CK2 off the copper-charged resin. Recombinant heterotetrameric CK2 was incubated with copper-charged resin and eluted with increasing concentrations of imidazole. Input represents 25% of total protein incubated with resin. Eluted protein was resolved by SDS-PAGE and assessed by immunoblotting with the indicated antibodies. **(C)** Quantification of the fold change in copper (parts per million) detected in recombinant CK2α preincubated with or without copper by ICP-MS (n = 2).

Given the enhanced enzymatic activity in the presence of copper, we hypothesized that CK2 directly binds to the metal. We used immobilized metal affinity chromatography (IMAC) to test this hypothesis. Recombinant heterotetrameric CK2 was incubated with uncharged resin or resin charged with various metals, and the extent of binding was assessed by elution of bound protein. Results of this experiment indicate that recombinant CK2 bound to the copper-charged resin but did not bind to the zinc- or iron-charged resins (Figure 3A). To validate the specificity of CK2 for the copper-charged resin, we conducted a competition experiment in which we assessed if CK2 could be eluted from the resin by titration of imidazole. Increasing concentrations of imidazole competed CK2 off the copper-charged resin (Figure 3B), demonstrating a specific interaction between CK2 and the copper-bound resin.

To further explore the physical capacity of CK2 to bind copper, we examined how incubating copper with the CK2α subunit influenced copper retention via inductively coupled plasma-mass spectrometry (ICP-MS). Purified CK2α was incubated with copper, excess copper was subsequently removed, and the protein was subjected to ICP-MS. The samples pre-incubated with copper demonstrated a three-fold increase in copper retention compared to control, although there was a baseline amount of copper already bound to purified CK2α prior to incubation (Figure 3C). Together, these results indicate that CK2, specifically the CK2α subunit, binds directly to copper *in vitro*.

### Met153 and His154 of CK2α Are Essential for Copper-Binding

**FIGURE 4:**
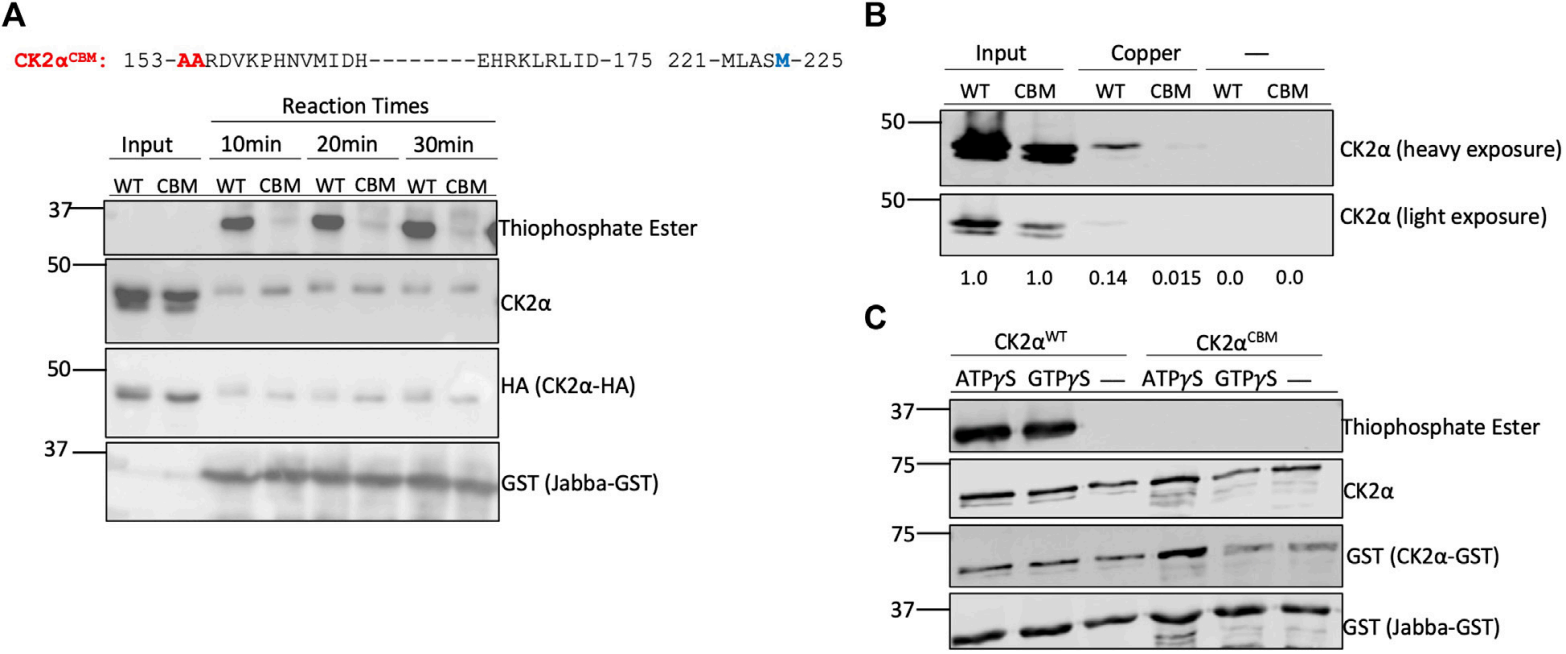
Met153 and His154 are essential for copper-binding. **(A)** Top: Sequence of human CK2α. Blue letters represent residues in CK2α that share homology with MEK1 at copper-binding sites. Red letters represent residues Met153 and His154 that were mutated to alanine to generate the CK2α^CBM^. Bottom: *in-vitro* kinase assay assessing CK2α^WT^ and CK2α^CBM^ enzyme activity. Constructs encoding HA-tagged CK2α^WT^ or CK2α^CBM^ were transfected into HEK293 cells. CK2α was then immunoprecipitated and incubated with GST-tagged Jabba and ATPγS. Following incubation with PNBM, reactions were resolved by SDS-PAGE and assessed by immunoblotting with the indicated antibodies. **(B)** Resin binding assay assessing the interaction of CK2α^WT^ and CK2α^CBM^ with copper-charged resin. Constructs encoding HA-tagged CK2α^WT^ or CK2α^CBM^ were transfected into HEK293 cells. Cell lysates were prepared and incubated with resin charged with or without copper. Input represents 25% total protein incubated with resin. Eluted protein was resolved by SDS-PAGE, assessed by immunoblotting with the indicated antibodies, and quantified. **(C)** *In-vitro* kinase assay assessing enzymatic activity of bacterially expressed CK2α^WT^ and CK2α^CBM^. Constructs encoding GST-tagged CK2α^WT^ or CK2α^CBM^ were transformed into bacteria. The purified kinases were then incubated with GST-tagged Jabba and ATPγS or GTPγS. Following incubation with PNBM, reactions were resolved by SDS-PAGE and assessed by immunoblotting with the indicated antibodies.

We next aimed to identify residues in CK2 that are critical for binding to copper. Here we focused on residues in MEK1 that were previously identified as being important for copper interaction and that shared substantial homology with the CK2α subunit (Figures 1A, 4A). As both MEK1 and CK2 are RD (arginine-aspartate) kinases containing an arginine immediately preceding the catalytic aspartate in the catalytic domain, we opted to mutate only the residues located directly adjacent to this region, Met153 and His154, due to their importance in regulating enzymatic activity in RD kinases (Johnson et al., 1996) (Figure 1A). To test if Met153 and His154 are indeed critical for copper-binding, we generated a mutant of CK2 (hereafter referred to as copper binding mutant, CBM) in which Met153 and His154 were mutated to alanine. We first assessed how the mutations influenced the catalytic activity of the CBM. We transiently expressed either HA-tagged CK2α^WT^ or CK2α^CBM^ in HEK293 cells and performed a subsequent immunoprecipitation (IP)-kinase assay. CK2α^WT^ or CK2α^CBM^ were immunoprecipitated using an anti-HA antibody and used in an *in vitro* kinase assay with Jabba as a substrate as previously described. While CK2α^WT^ was able to phosphorylate Jabba, the CK2α^CBM^ did not and thus appeared to be catalytically compromised (Figure 4A). To confirm that CK2α^CBM^ was not catalytically inactive due to misfolding induced by the mutations, we assessed the association of CK2α^WT^ and CK2α^CBM^ with the CK2β subunit by co-immunoprecipitation analysis. HA-tagged CK2α^WT^ and CK2α^CBM^ were immunoprecipitated, and the immunoprecipitates were probed with an anti-CK2β antibody. Results from these experiments showed that both WT and CBM bind the CK2β subunit equally (Supplemental Figure S5), indicating that the mutations do not interfere with the interaction between CK2α and CK2β and suggest the heterotetramer is properly assembled.

We next tested if the CK2α^CBM^ had a reduced ability to bind to copper using IMAC. Lysates from HEK293 cells transiently expressing CK2α^WT^ or CK2α^CBM^ were incubated with the (IMAC) copper-charged resin. Protein bound to the resin was eluted to assess the extent of binding, and the eluates were probed for CK2α. CK2α^CBM^ demonstrated a reduction in its capacity to bind the copper-charged resin compared to CK2α^WT^ (Figure 4B). To further validate the influence of the mutations on CK2 enzymatic activity, we expressed and purified GST-tagged CK2α^WT^ and CK2α^CBM^ from bacteria and performed an *in vitro* kinase assay using GST-tagged Jabba as a substrate. Results from this experiment corroborated that CK2α^CBM^ appears to be catalytically impaired (Figure 4C). Collectively, these results indicate that residues Met153 and His154 are vital for copper binding and the enzymatic activity of CK2α.

### Copper Modulates CK2 Kinase Signaling *in vivo*

**FIGURE 5:**
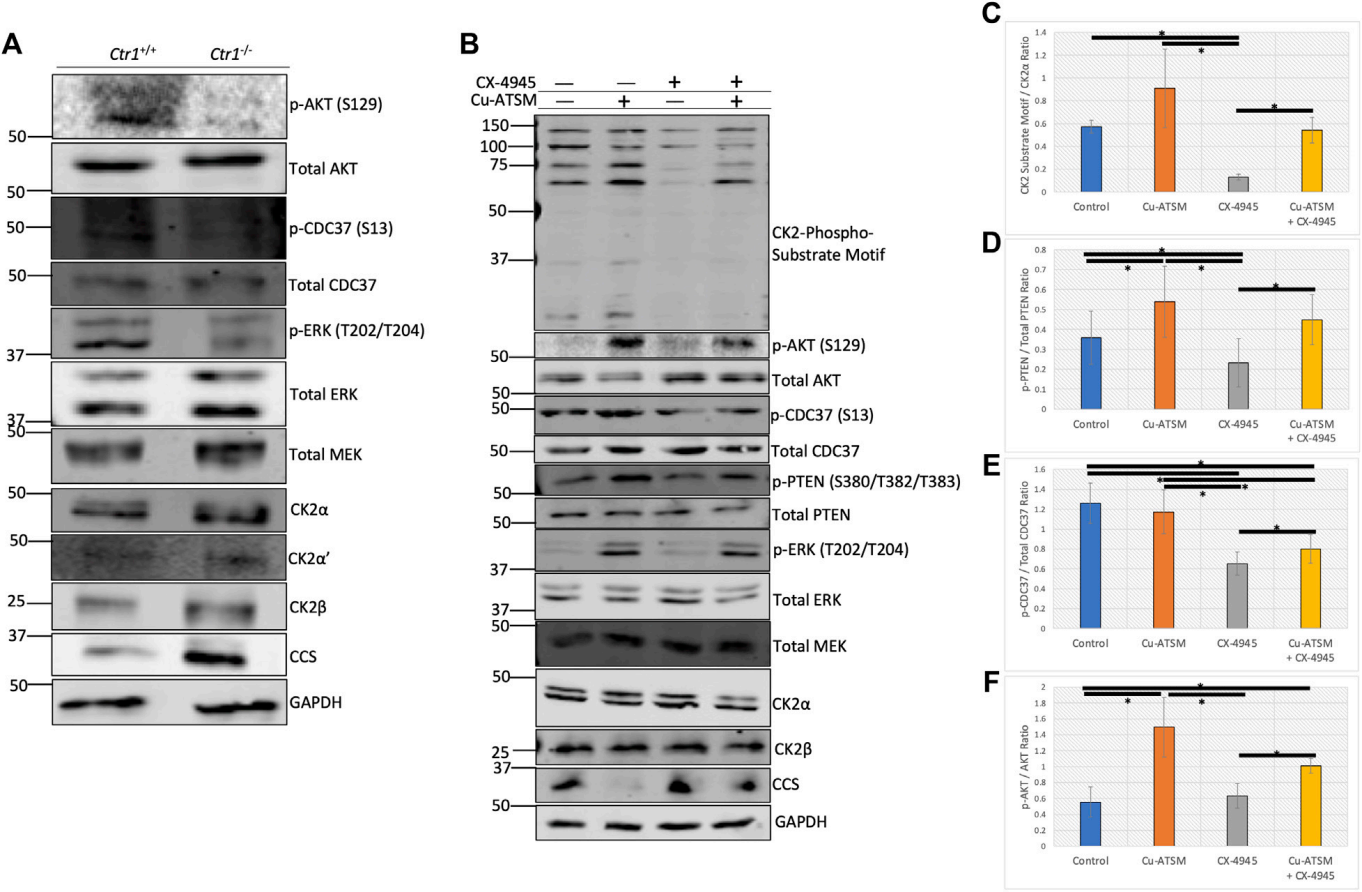
Copper modulates cellular CK2 kinase signaling. **(A)** Lysates from *Ctr1* ^+/+^ and *Ctr1*−/− MEFs were resolved by SDS-PAGE and assessed by immunoblotting with the indicated antibodies. **(B)** Lysates from HeLa cells treated with or without Cu-ATSM and/or CX-4945 were resolved by SDS-PAGE and assessed by immunoblotting with the indicated antibodies.**(C)** Quantification of the CK2-phospho-substrate to CK2α ratio by immunoblot in panel B (n = 4). Data are represented as mean fold change ±SEM. *indicates *p* < 0.05 as calculated by a paired student *t*-test. **(D)** Quantification of the p-PTEN (S380/T382/T383) to total PTEN ratio by immunoblot in panel B (n = 4). Data are represented as mean fold change ±SEM. *indicates *p* < 0.05 as calculated by a paired student *t*-test. **(E)** Quantification of the p-CDC37 (S13) to total CDC37 ratio by immunoblot in panel B (n = 4). Data are represented as mean fold change ±SEM. *indicates *p* < 0.05 as calculated by a paired student *t*-test. **(F)** Quantification of the p-AKT (S129) to total AKT ratio by immunoblot in panel B (n = 4). Data are represented as mean fold change ±SEM. *indicates *p* < 0.05 as calculated by a paired student *t*-test.

To support the findings of the *in vitro* experiments, we next investigated the effect of copper on CK2 signaling in cells. We first utilized *Ctr1* ^*−/−*^ MEFs to evaluate whether loss of copper import influenced phosphorylation of CK2-specific substrates. We validated the reduction of intracellular copper in the C*tr1* ^*−/−*^ MEFs via the reduction in phosphorylation of ERK by MEK (Brady et al., 2014; Turski et al., 2012), a copper-dependent kinase, and by the increase in the expression of the copper chaperone for superoxide dismutase (CCS), which is an indirect readout for copper status: its expression is inversely proportional to intracellular copper levels (Figure 5A) (Bertinato and L'Abbé, 2003). Lysates from WT and *Ctr1* ^*−/−*^ MEFs were probed with antibodies to assess CK2 signaling. We found that Ctr1 knockout resulted in a reduction in site-specific phosphorylation of two CK2 substrates, AKT at Ser129 and CDC37 at Ser13 (Miyata & Nishida, 2004; Di Maira et al., 2005), indicating that a reduction in intracellular copper concentration reduces CK2 kinase activity (Figure 5A). Importantly, there were no observed changes in protein expression of CK2α, CK2α′, or CK2β, that could account for these signaling differences.

We next employed a pharmacological approach to increase levels of intracellular copper. In addition to incubating cells with excess copper, we also used a copper ionophore delivery system, namely diacetyl-bis(4-methyl-3-thiosemicarbazonato) copper II (Cu-ATSM). Importantly, there is evidence that Cu-ATSM facilitates the delivery of copper to cupro-enzymes, such as SOD1, by increasing the pool of metallated protein (Roberts et al., 2014). We first verified that CK2 activity is enhanced *in vitro* by Cu-ATSM (Supplemental Figures S6, 7), similar to incubation with copper using both Jabba and AKT as substrates. To assess the efficacy of the copper delivery methods, we then treated serum-starved HeLa cells with serum, copper, or Cu-ATSM, and then analyzed changes to the copper-modulated MAPK pathway. Following treatments, cell lysates were assessed by immunoblotting. We observed a slight increase in ERK phosphorylation in response to serum and copper, whereas Cu-ATSM induced a dramatic increase in phosphorylation of ERK (Supplemental Figure S8). In addition, Cu-ATSM was able to induce an increase in CK2-specific phosphorylation of AKT at Ser129 and CDC37 at Ser13 (Miyata & Nishida, 2004; Di Maira et al., 2005), as well as slightly increase the intensity of the CK2-substrate motif antibody, which is used as a global CK2 phosphorylation readout (Supplemental Figure S8).

Given the observed increase in CK2 substrate phosphorylation upon treatment with Cu-ATSM, we next wanted to test if we could modulate these Cu-ATSM-induced changes with the ATP-competitive CK2 inhibitor CX-4945 (silmitastertib). We pre-incubated serum-starved HeLa cells with CX-4945 to ensure inhibition of CK2 prior to Cu-ATSM delivery. Following treatments, cell lysates were prepared and assessed by immunoblotting. As previously observed, treatment with Cu-ATSM increased site-specific phosphorylation of AKT at Ser129 and CDC37 at Ser13, as well as the signal obtained with the CK2-substrate motif antibody and phosphorylated PTEN at Ser380/Thr382/Thr382, an additional CK2-specific substrate (Torres & Pulido, 2001) (Figure 5B). As expected, treatment with CX-4945 resulted in a reduction in phosphorylation of all CK2 substrates analyzed, with the exception of AKT that, at baseline, showed no detectable phosphorylation (Figure 5B). Interestingly, the combination treatment of CX-4945 and Cu-ATSM demonstrated that the reduction seen by CX-4945 could be partially rescued by the treatment of Cu-ATSM on all of the CK2 substrate readouts (Figure 5C–F). We corroborated these findings in two other cancer cell lines, U87-MG glioblastoma cells and A375 melanoma cells (Supplemental Figures S9, S10). Together, these results indicate that CK2-dependent signaling pathways are modulated by copper availability.

## Discussion

Despite the long-held notion that copper is predominantly a static inorganic co-factor, there is now substantial evidence that copper plays a more dynamic role within the cell. Copper has been shown to regulate the activity of multiple kinases, including MEK1, ULK1, and PDK1 (Brady et al., 2014; Tsang et al., 2020; Guo et al., 2021), and here we demonstrate that copper also regulates CK2. Specifically, we demonstrate that copper enhances CK2 activity and that this effect can be abolished using a copper-specific chelator. We also showed that CK2 directly binds to copper and identified residues critical for copper-binding. Finally, we demonstrated that decreases in intracellular copper reduce CK2 signaling, whereas increases in intracellular copper increase CK2 signaling.

While copper enhances CK2 activity, the conditions under which this occurs are dependent on the redox state of copper. Indeed, cuprous copper is the predominant intracellular form of copper, and it has been shown that MEK and ULK also require reduced copper for catalytic enhancement. Our ICP-MS data demonstrate that a portion of purified CK2α was copper-bound prior to incubation with additional copper, indicating a potential for reduced capacity for further copper interaction and, therefore, enzymatic enhancement (Figure 3C). While not directly tested, we further speculate that copper would also enhance the catalytic activity of CK2α′ based on homology of predicted copper-binding residues between CK2α and CK2α’.

Understanding the physiological role of copper on kinase activity has the added intricacy of determining the cellular mechanism of copper delivery. A recent study established that CCS delivers copper to MEK1, thereby modulating the signaling activity of MEK1 (Grasso et al., 2021). However, the copper delivery mechanism for CK2 is unknown, and whether CCS serves in this capacity or whether another chaperone is involved has yet to be determined. This study also identified two residues essential for copper-binding, Met153, and His154. However, these residues are conserved in many kinases across the kinome. Therefore, we cannot discount the influence of these mutations as being potentially copper-independent. Future studies will be directed at systematically identifying all putative copper-binding residues using an unbiased approach.

Two findings emerged from our work suggesting that copper-mediated regulation of CK2 may be more nuanced than simple enhancement of enzymatic activity. The first was that the enhancement in substrate phosphorylation by CK2 in the presence of copper only occurred using ATP, whereas there was no apparent enhancement with GTP (Supplemental Figures S2, S3). Although CK2 exhibits dual co-substrate specificity, to our knowledge, this is the first evidence suggesting that nucleotide-specific regulation may exist (Niefind et al., 1999). The second intriguing finding was the observation of differential banding patterns in lysates from the Ctr1 wildtype versus null MEFS when probed with the CK2-substrate motif antibody (Figure 1C). Without knowing the identity of the specific proteins corresponding to the bands, we cannot discount changes in protein abundance due to the presence or absence of Ctr1. However, both of these findings suggest a potential underlying complexity in the enhancement of CK2 activity by copper that warrants further exploration.

Since CK2 plays a critical role in the progression of multiple types of cancer and other diseases, there has been significant interest in CK2 inhibition in the clinical setting, highlighted by several clinical trials with the CK2 inhibitor CX-4945 (clinicaltrials.gov ID# NCT02128282, NCT03897036, NCT01199718, NCT03904862, NCT00891280, NCT04668209). With inhibition as the targeted outcome, it is critical to understand any potential mechanisms that might modulate the effect of the inhibitor in a cellular context. Here we report that copper enhances CK2 activity and that treatment with Cu-ATSM appears to partially restore CK2 activity following CX-4945 treatment. These data suggest that copper may modulate the efficacy of CX-4945, suggesting the potential benefit of combining CX-4945 with copper chelation therapy to achieve the best outcome.

## Data Availability

The original contributions presented in the study are included in the article/Supplementary Material, further inquiries can be directed to the corresponding author.
