# Supplementary figures and images for: Copper Modulates the Catalytic Activity of Protein Kinase CK2
Source: Front Mol Biosci. 2022 Jun 9;9:878652. doi: 10.3389/fmolb.2022.878652 (PMC9224766; doi:10.3389/fmolb.2022.878652)

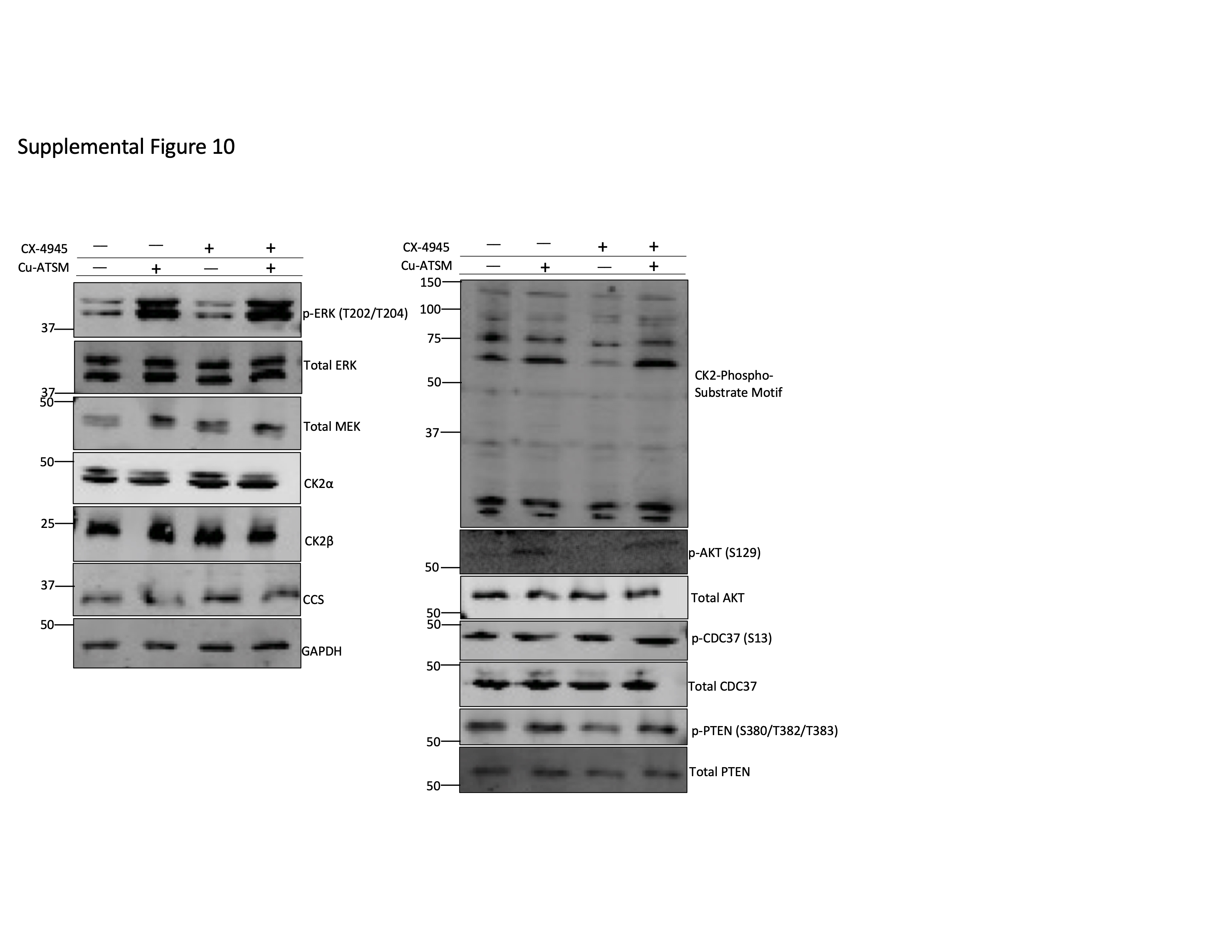

Supplement: Supplementary file 1 [file Image10.jpg]

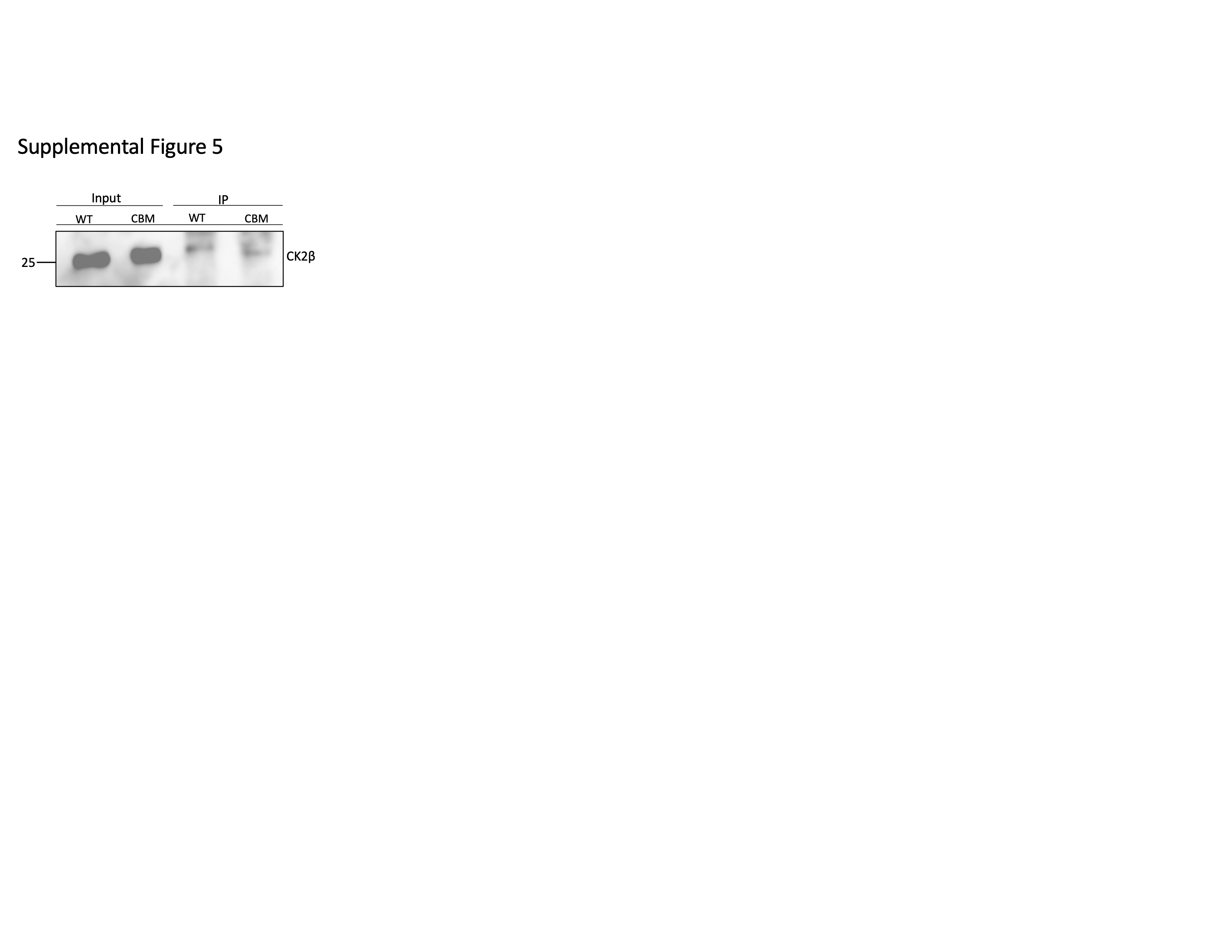

Supplement: Supplementary file 2 [file Image5.jpg]

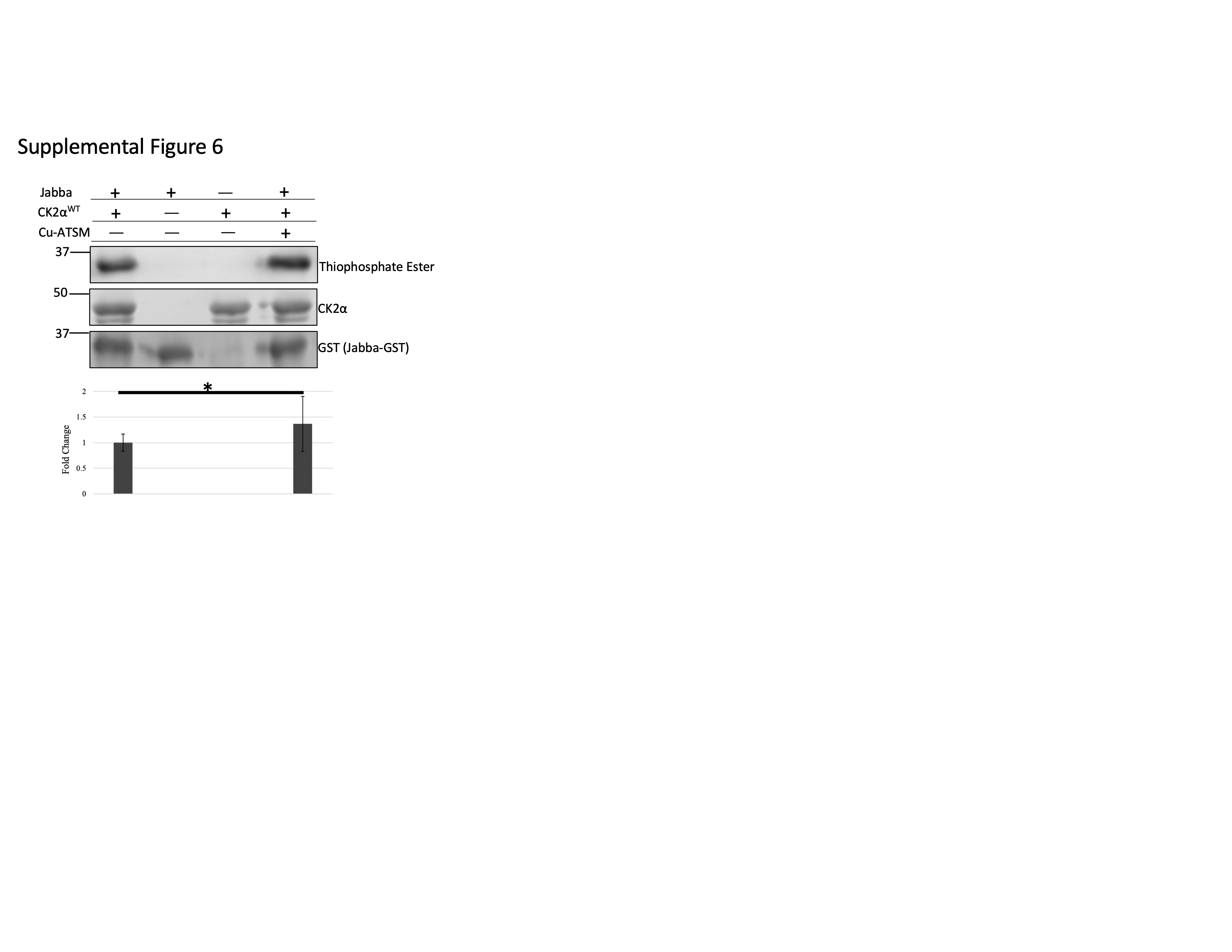

Supplement: Supplementary file 3 [file Image6.jpg]

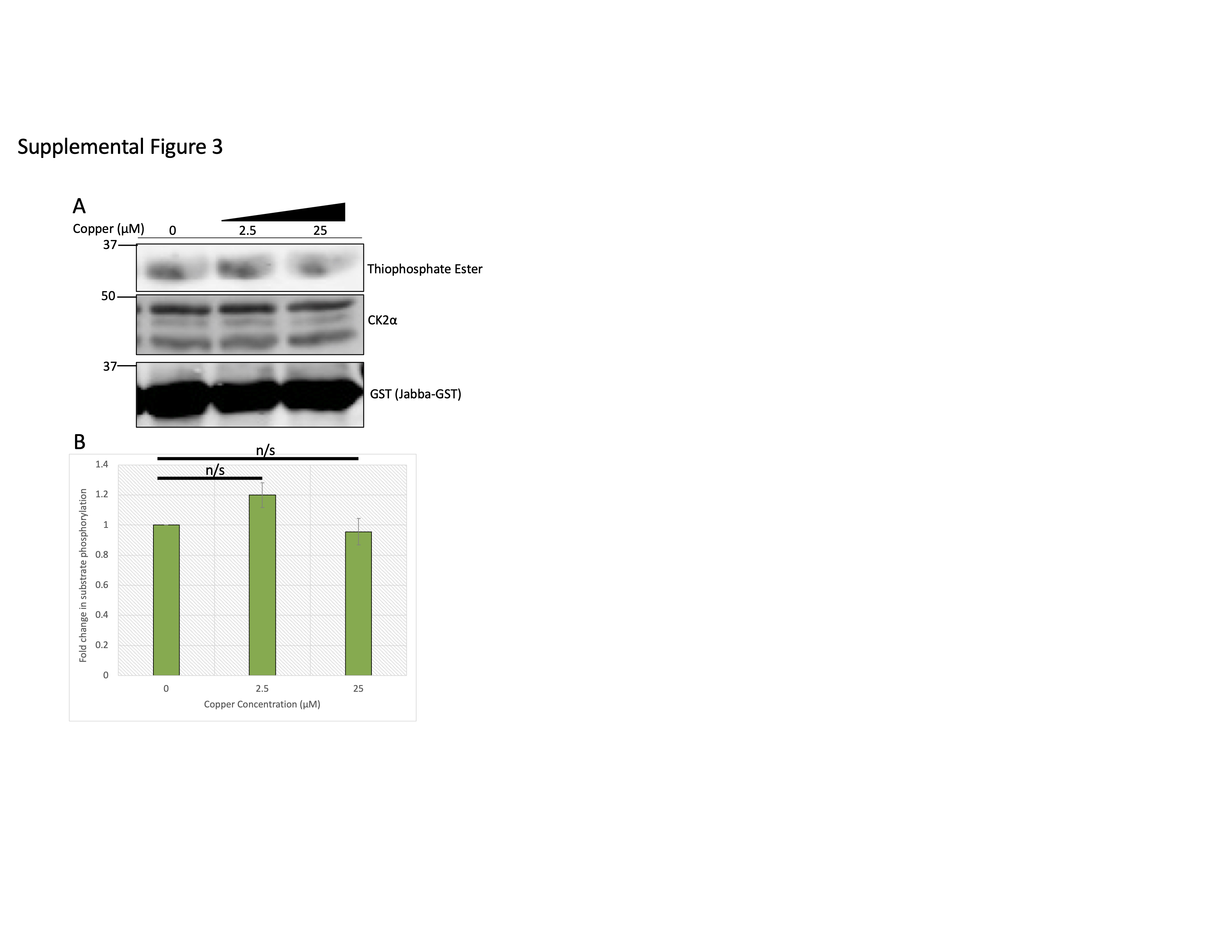

Supplement: Supplementary file 4 [file Image3.jpg]

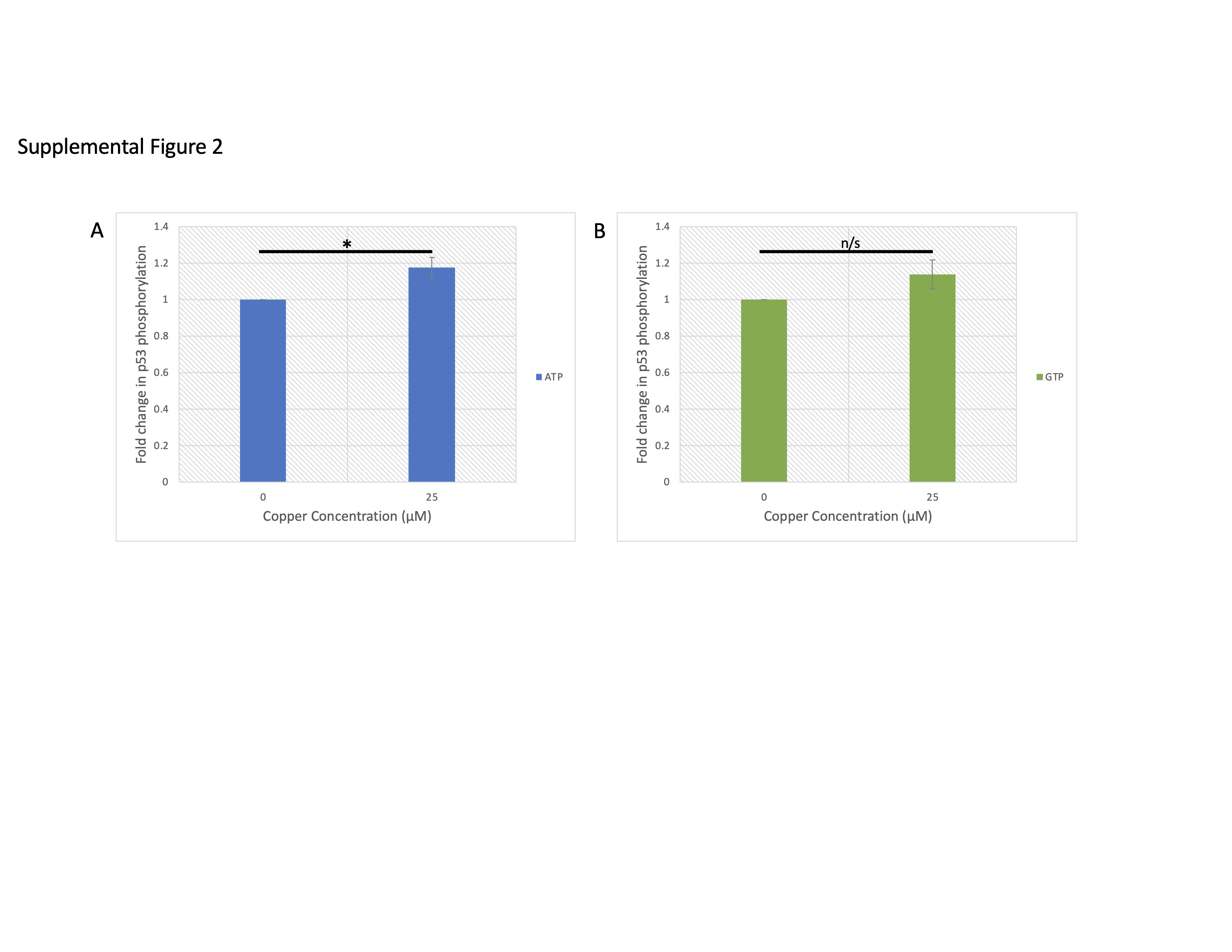

Supplement: Supplementary file 5 [file Image2.jpg]

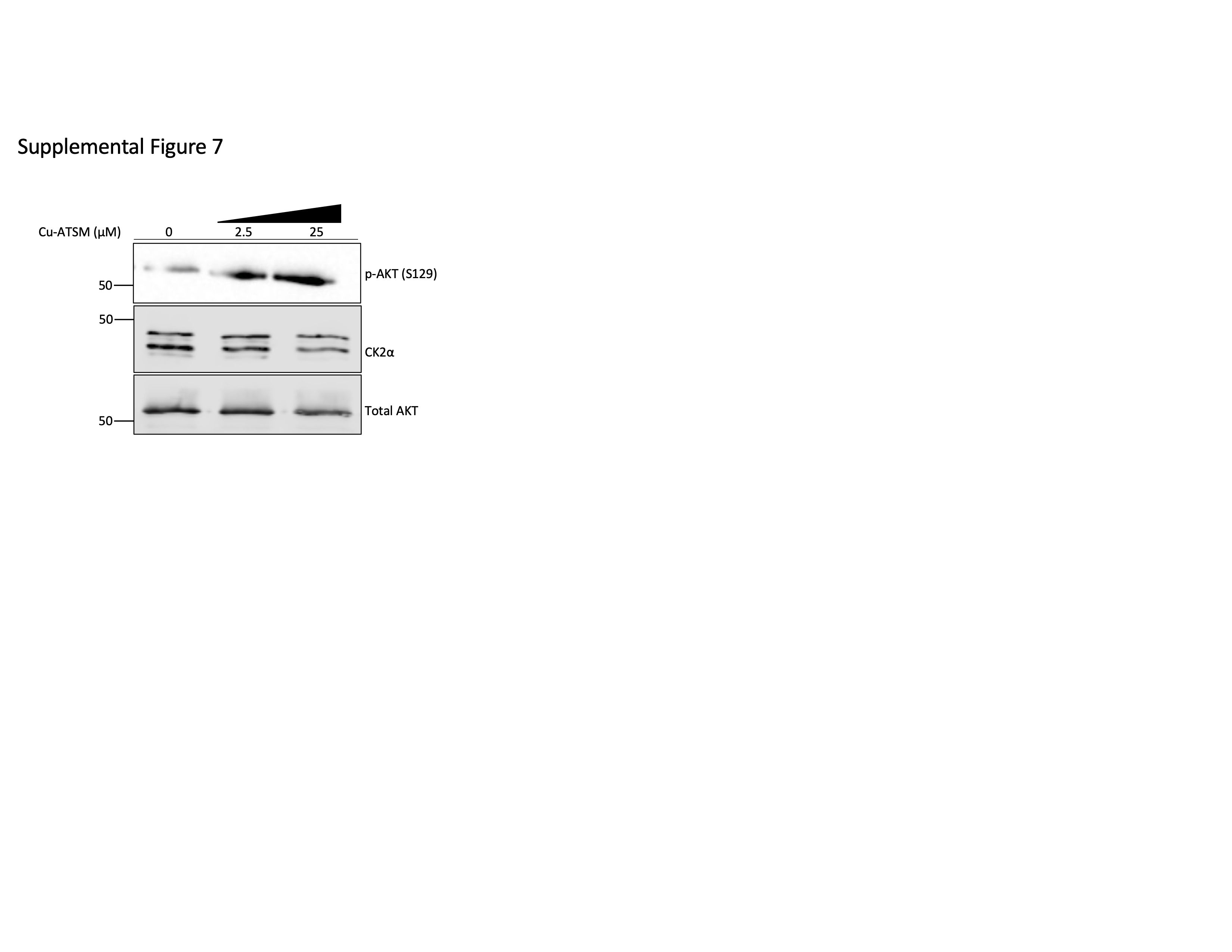

Supplement: Supplementary file 6 [file Image7.jpg]

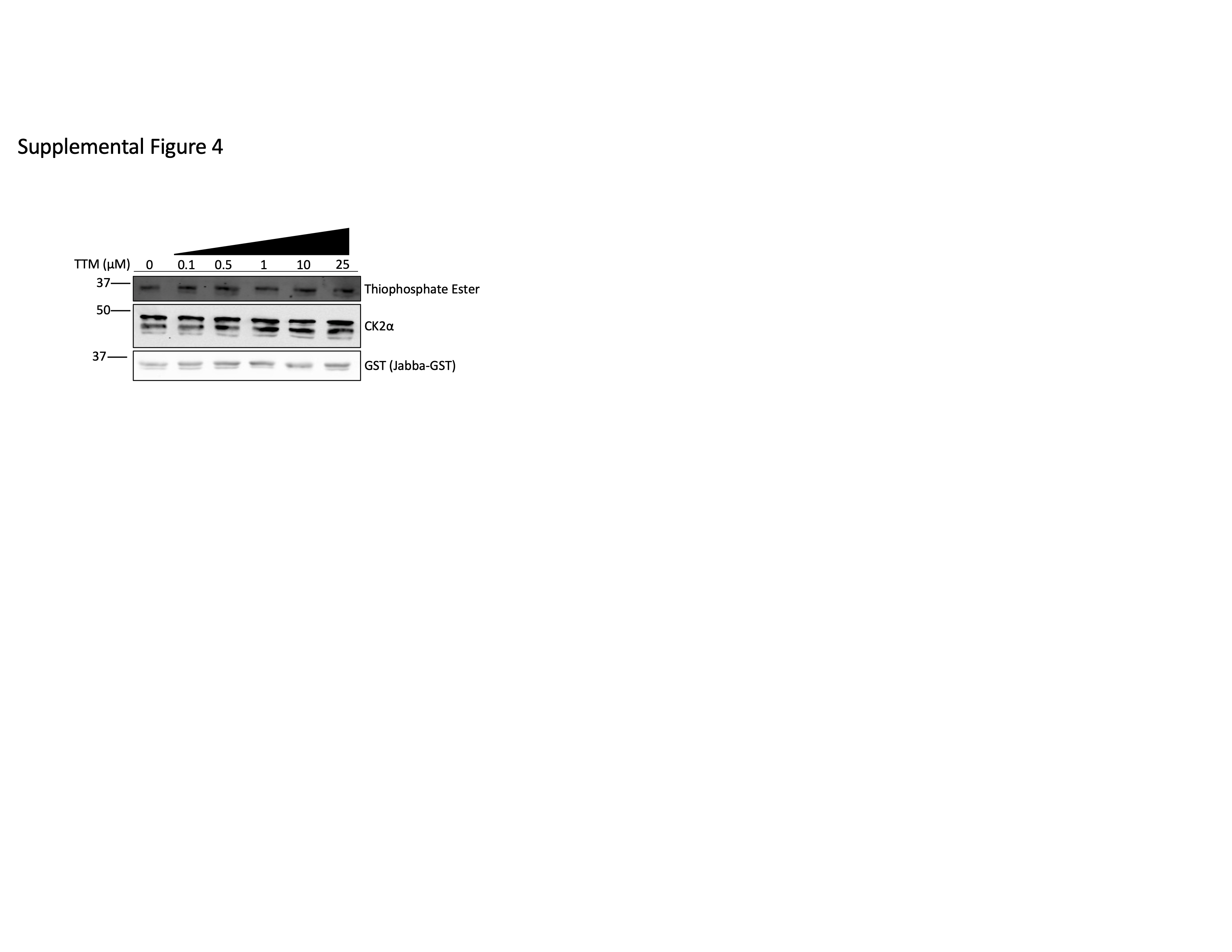

Supplement: Supplementary file 7 [file Image4.jpg]

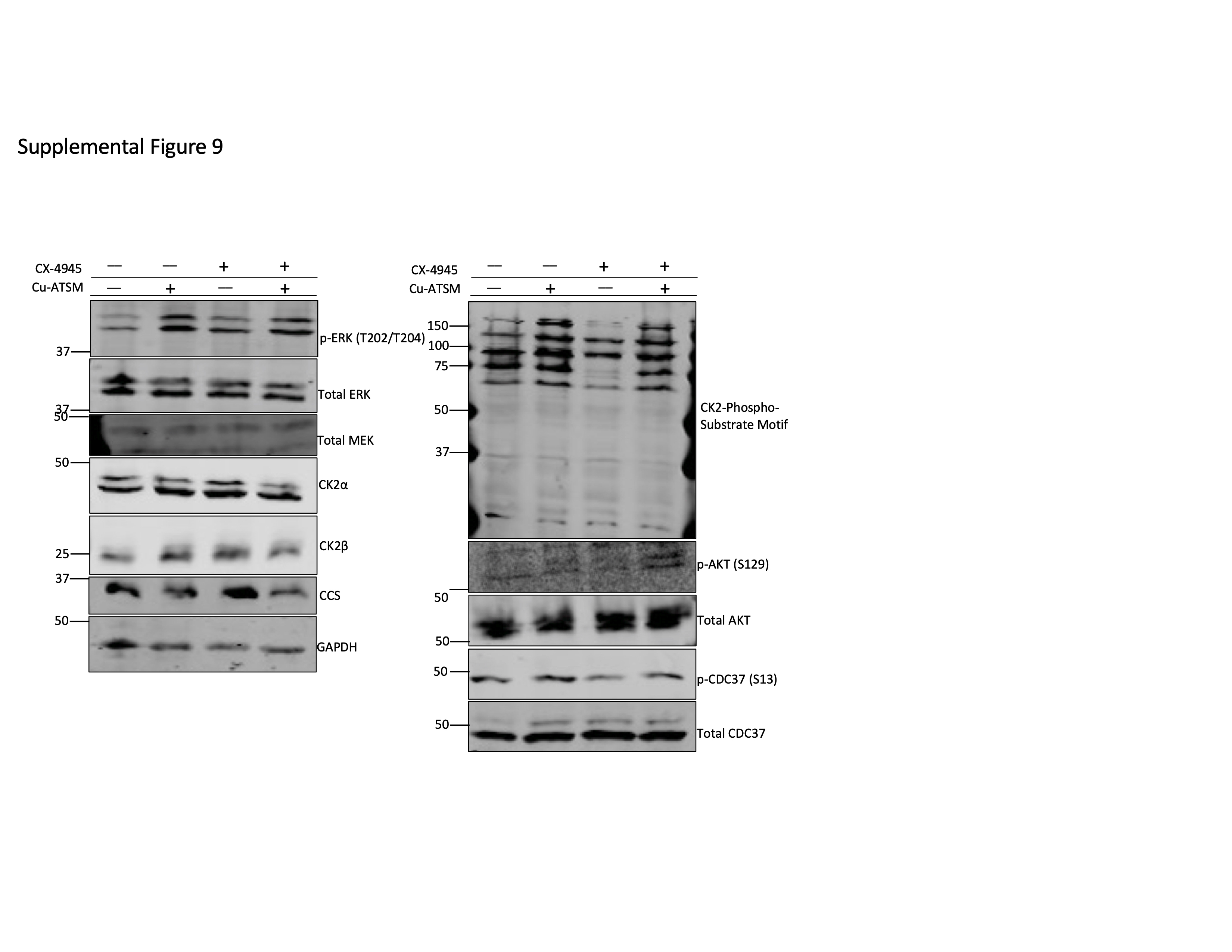

Supplement: Supplementary file 8 [file Image9.jpg]

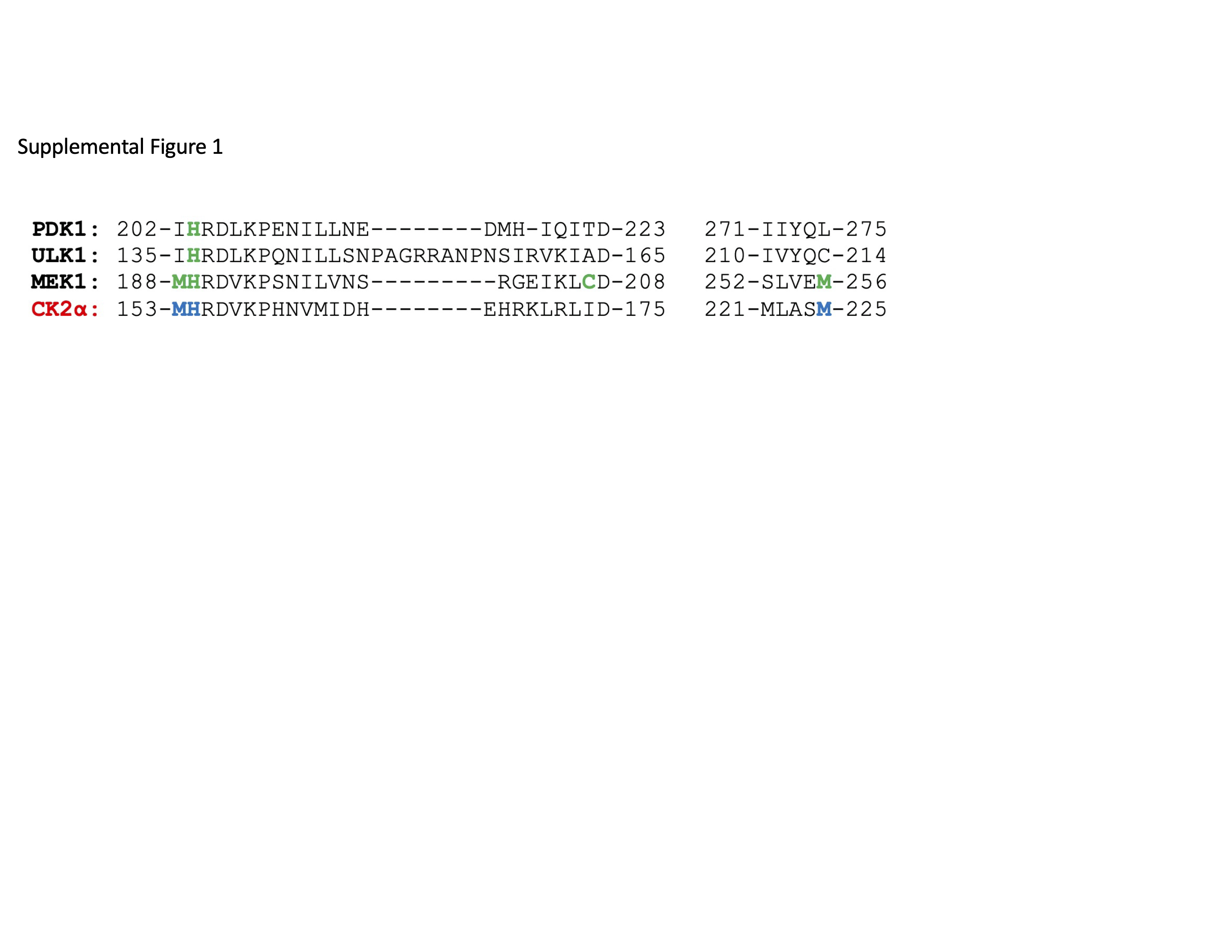

Supplement: Supplementary file 9 [file Image1.jpg]

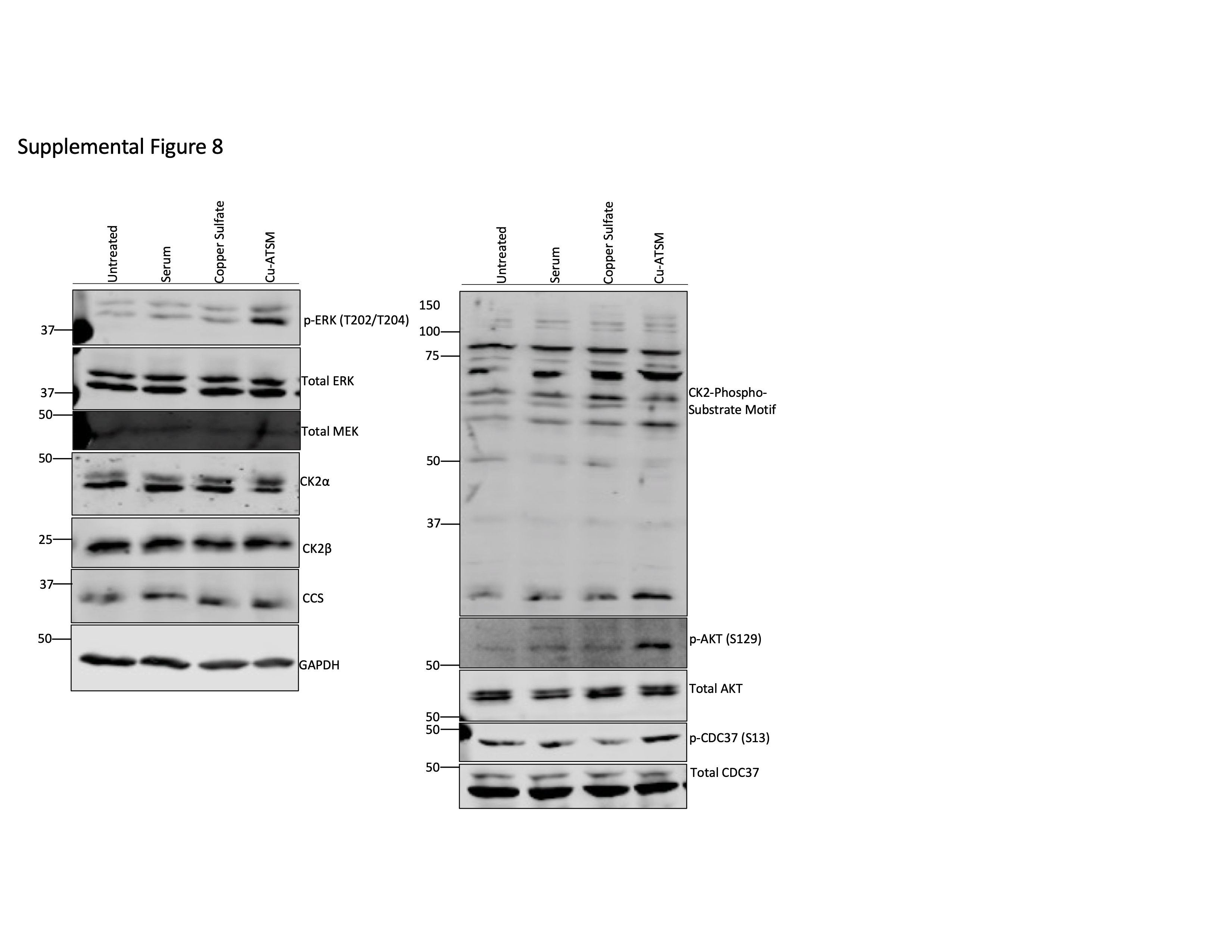

Supplement: Supplementary file 10 [file Image8.jpg]
